# Development and validation of ester impregnated pH strips for locating nasogastric feeding tubes in the stomach—a multicentre prospective diagnostic performance study

**DOI:** 10.1186/s41512-021-00111-9

**Published:** 2021-12-14

**Authors:** Melody Ni, Mina E. Adam, Fatima Akbar, Jeremy R. Huddy, Simone Borsci, Peter Buckle, Francesca Rubulotta, Reuben Carr, Ian Fotheringham, Claire Wilson, Matthew Tsang, Susan Harding, Nichola White, George B. Hanna

**Affiliations:** 1 Department of Surgery and Cancer, Imperial College London, Academic Surgical Unit, 10th Floor, QEQM Building, St. Mary’s Hospital, London, W2 1NY UK; 2 Cognitive Psychology and Ergonomics, University of Twente, Enschede, The Netherlands; 3 Imperial College Healthcare NHS Trust, London, UK; 4 Ingenza Ltd., Roslin, UK; 5 The Royal Bournemouth and Christchurch Hospitals NHS Foundation Trust, Bournemouth, UK; 6 Medway Maritime NHS Foundation Trust, Gillingham, UK

**Keywords:** Diagnostics, Nutrition, NG-tubes, Health economics, Adoption barriers

## Abstract

**Background:**

NG (nasogastric) tubes are used worldwide as a means to provide enteral nutrition. Testing the pH of tube aspirates prior to feeding is commonly used to verify tube location before feeding or medication. A pH at or lower than 5.5 was taken as evidence for stomach intubation. However, the existing standard pH strips lack sensitivity, especially in patients receiving feeding and antacids medication. We developed and validated a first-generation ester-impregnated pH strip test to improve the accuracy towards gastric placements in adult population receiving routine NG-tube feeding. The sensitivity was improved by its augmentation with the action of human gastric lipase (HGL), an enzyme specific to the stomach.

**Methods:**

We carried out a multi-centred, prospective, two-gate diagnostic accuracy study on patients who require routine NG-tube feeding in 10 NHS hospitals comparing the sensitivity of the novel pH strip to the standard pH test, using either chest X-rays or, in its absence, clinical observation of the absence of adverse events as the reference standard. We also tested the novel pH strips in lung aspirates from patients undergoing oesophageal cancer surgeries using visual inspection as the reference standard. We simulated health economics using a decision analytic model and carried out adoption studies to understand its route to commercialisation. The primary end point is the sensitivity of novel and standard pH tests at the recommended pH cut-off of 5.5.

**Results:**

A total of 6400 ester-impregnated pH strips were prepared based on an ISO13485 quality management system. A total of 376 gastric samples were collected from adult patients in 10 NHS hospitals who were receiving routine NG-tube feeding. The sensitivities of the standard and novel pH tests were respectively 49.2% (95% CI 44.1‑54.3%) and 70.2% (95% CI 65.6‑74.8%) under pH cut-off of 5.5 and the novel test has a lung specificity of 89.5% (95% CI 79.6%, 99.4%). Our simulation showed that using the novel test can potentially save 132 unnecessary chest X-rays per check per every 1000 eligible patients, or direct savings of £4034 to the NHS.

**Conclusions:**

The novel pH test correctly identified significantly more patients with tubes located inside the stomach compared to the standard pH test used widely by the NHS.

**Trial registration:**

http://www.isrctn.com/ISRCTN11170249, Registered 21 June 2017—retrospectively registered

**Supplementary Information:**

The online version contains supplementary material available at 10.1186/s41512-021-00111-9.

## Introduction

Nasogastric tubes (NG-tubes) are a common means of providing nutrition and medication for patients who are malnourished or at risk of malnutrition [1]. National clinical guidelines recommend using a combination of non-radiographic and radiographic methods to verify tube location before feeding or medication to prevent adverse events due to respiratory placements [2]. Of the non-radiographic methods, pH testing is the preferred first-line test. The UK National Patient Safety Agency has recommended testing the pH of tube aspirates before every feed and at least once daily to confirm gastric placement [3, 4]. Feeding is considered safe when an aspirate of pH between 1 and 5.5 has been established, which indicates acidity and therefore is associated with stomach intubation. When the pH is higher than 5.5, a chest radiograph is recommended to verify correct tube placement before feeding can take place [5].

Despite these precautions, reported rates of tube misplacements on insertion and tube migration after correct initial placement vary between 1.3 and 50% in adults [6]. Adverse feeding incidents, which NHS England has classified as a ‘Never Event’ [7], continue to be reported despite the precautions. Between September 2011 and March 2016, 95 feeding incidents were documented, including 32 deaths [8]. A recent study showed that the sensitivity of pH ≤ 5.5 in correctly identifying gastric samples was 68% (95% CI 57 to 77%) and the specificity was 79% (95% CI 74 to 84%) in adult patients using samples taken during gastroscopy and bronscopy [9]. Gastric pH is elevated in patients who are receiving feeding and/or acid suppression medication (such as proton pump inhibitors) [10, 11]. Patients are sent for chest X-rays (CXR) despite having the tube correctly placed inside the stomach. Reducing the pH cut-off was not recommended since this would increase the demand for CXRs [5]. In addition to being far more expensive than pH strips, waiting for CXRs could potentially delay feeding up to 47 h [12]. CXRs are also subject to user interpretation errors, leading to feeding incidents [13]. Sorokin and Gottlieb [14], for instance, observed a tube misplacement rate of 2.4% (50/2079 tubes) using relevant chest radiograph reports to identify tube placements. In this series, 26 out of 50 patients with respiratory misplacement were mechanically ventilated. For differentiating between positioning in the stomach, lung and oesophagus, pH testing remains the safest and the most accessible amongst available bedside tests, including auscultation, colorimetric and magnetic guidance [15, 16]. The current pH tests are also cost-effective for the NHS [17].

We developed a novel ester impregnated pH strips (Ingenza Ltd., Scotland, UK) [18] to improve accuracy of pH testing in identifying stomach tube placements. The novel strips exploited the ester-hydrolysis reaction catalysed by *Human Gastric Lipase* (HGL), an enzyme unique to the stomach. This reaction generates acid in situ from a pH strip impregnated with an ester substrate, such as tributyrin, thus augmenting the diagnostic accuracy of the conventional bedside pH test [12]. In a pilot study using samples collected from surgical patients being treated at St Mary’s Hospital in London, the ester-impregnated strips correctly identified 97.2% of the 36 gastric samples, as having a readout of pH between 1 and 5.5, compared to 65.7% identified using the standard pH test. Both the HGL and standard pH strips were able to identify all 23 lung samples as having a pH higher than 5.5, thus achieving 100% specificity under the recommended cut-off [19]. Human factors research informed the development of the novel strips [12].

The primary aim of this study was to develop and validate at scale a first-generation ester impregnated pH strip test for locating blindly inserted NG-tubes in adult patients. The secondary aim was to assess cost savings due to a reduction in CXR demand and the route to adoption of the novel strips.

## Materials and methods

### Assay development, validation and quality control

The ester impregnated pH papers utilise Human Gastric Lipase (HGL) enzyme, shown to be present only in the stomach [20], by exploiting its mode of biochemical action to hydrolyze a tributyrin (ester) substrate impregnated onto standard pH paper . When HGL is present in the acquired bedside aspirate, butyric acid is released following HGL-mediated hydrolysis of tributyrin, increasing local acidity which in turn is detectable by the modified pH strip. Therefore, the ester impregnated pH strip is able to utilise and detect both gastric hydrochloric acid (HCl) and/or HGL biomarker activities. This dual-marker approach greatly increases the sensitivity of the novel test compared to the incumbent pH papers. Assay validation was conducted in compliance with ISO13485 Medical Devices, and under control of Ingenza’s Quality Management System. Validation was carried out using representative microbial and porcine pancreatic lipases and then on patient samples (from a representative pool of patients receiving various medications, including antacids and proton pump inhibitors (PPIs)) for further improvement. Limits of detection, sensitivity and stability were determined. The final formulation was proven and validated to be suitably sensitive and specific towards both gastric biomarkers (HCl and HGL) and within clinically acceptable assay reading times when concentrations are within the normal range found in gastric aspirate.

Standard pH test strips (GBUK/Enteral) were purchased and impregnated with a solution containing 1% tributyrin. Tributyrin impregnation was carried out upon individual batches of 400 pH strips, which were then dried, packaged in 100 strip lots and stored at ambient temperature. Quality control was carried out by a random sample of 5 strips from each batch preparation and tested for their response to a solution of *Porcine pancreatic* lipase (PPL) at pH 7.0. Following 2-min incubation, all strips were read by two operators (See Additional files for full protocol details).

### Diagnostic accuracy study

We reported the diagnostic accuracy study in line with the STARD guidelines [21, 22].The study is a prospective, observational, cross-sectional, multi-centre and two-gate diagnostic accuracy study which compared the accuracy of the novel ester impregnated pH test (novel pH test) in detecting stomach aspirates versus the standard pH test paper (standard pH test) supplied by GBUK Enteral Ltd. (UK), which is the largest distributor in Europe, as well as a major NHS supplier. Two reference tests were used. The first was a CXR if requested as part of standard patient care as per clinical guideline when the pH of tube was found to be greater than 5.5. In the absence of a CXR, clinical observation of any signs or indication which might lead to suspicion of adverse events, such as signs of agitation and difficulty in breathing, during the course of the study, was used as evidence that the tube had been safely placed inside the stomach. The study protocol (IRAS 192968) is available at http://www.isrctn.com/ISRCTN11170249.

We included any patients who required the insertion of NG-tubes for supplementary enteral feeding as part of their clinical management. We excluded patients who were under the age of 18 years, prisoners or patients sectioned under the UK Mental Health Acts. Patients who were unable to consent were eligible, as long as consent was given by their legal guardians. We engaged consecutive patients until the recruitment target was fulfilled.

After a tube was inserted and aspirated, the researcher applied tube aspirate to both the standard and the novel strips. If no aspirates were available, the tube was adjusted and another attempt was made to aspirate. Starting with the standard strip, the researcher correlated and silently read the corresponding pH value from the calibration colour chart provided on the pH strip container. The researcher then asked a colleague, blinded to this first reading, to make a second independent reading of the standard strip against the same calibration chart. The researcher then photographically documented the developed strip and colour chart with the patient number and date of sample collection. The same procedure was repeated for the novel test. The sequence of testing, i.e. standard followed by novel strips, was chosen to ensure that sufficient time had passed to allow the lipase catalysed reaction in the novel strip to reach completion. Although this would be expected within 1 min, the same as the standard strips, we recommended 2 min in the clinical study and tested the acceptance of the longer waiting time. The researchers logged all readings in a standardised data collection form that was uploaded to a centralised secure database hosted at Imperial College. The default tube location was inside the stomach. The researcher was required to update the tube sites either if adverse events following tube insertion had indicated misplacements or if alternative sites were confirmed by chest X-rays.

In addition to the primary St. Mary’s hospital site, the study was adopted by 9 other hospitals through the NIHR Clinical Research Network (CRN) (Additional files). Each adoption site assigned a dedicated researcher (either a nurse or a research practitioner) to be responsible for local patient recruitment, data collection and management. They were given a half-day face to face training by the research team (FA, SB) at Imperial College. Researchers (MN, FA, MA) reviewed the first 5 samples at each site to ensure quality in data collection. The photographs of the samples were retrieved and reviewed to ensure accuracy in pH readings using both the novel and the standard strips. Any issues identified were communicated to the local researchers from the adoption site; advice was given for improvement. A follow-up check of further 5 samples was carried out to ensure the practice had improved. This procedure enabled us to identify several issues in data collection during the early phase of the study such as inadequate or unequal volumes of aspirate being applied to either or both strips and insufficient time being allowed to lapse prior to strip readings. These findings led to a period of troubleshooting whilst we suspended the trial to investigate the causes. Eventually the issues were resolved; the modified study protocol was approved and implemented, and the trial was re-enacted after 6 weeks (Additional files). The team who developed the novel pH test did not take part in the conduct or the analysis of the diagnostic accuracy of clinical study.

In parallel with the diagnostic accuracy study, which aimed at stomach placements, we (MA, GH) collected lung aspirates from patients who were intubated for elective surgery for gastro-oesophageal cancer resection operations. Tube placement in the lung was confirmed by the anaesthetist using capnography. Each aspirate was subsequently tested on the novel ester impregnated strips following the same procedure implemented in the gastric study. We did not test lung aspirates on standard pH strips, as these had been extensively researched [11, 23].

### Cost-saving assessment

We built a decision analytic model to simulate potential radiography cost savings to the NHS, based on NHS reference costs and applying an annual inflation rate of 3.5% to bring the costs up to date. We assumed that the novel strips would replace the standard pH strips as the first-line test for locating NG tubes, and used the sensitivity data from the clinical study, assuming that the standard and novel test had identical specificities. Since the two strips had an identical design, we assumed that the new strip would not require modifications of the existing pathway. We modelled the population with nasogastric tubes already placed but allowed the possibility that tube aspirates might be unsuccessful. CXRs were recommended when a pH reading greater than 5.5 had been confirmed. In addition to the standard scenario, we considered a *recheck* scenario where a further pH test was carried out and CXRs were only requested if a second pH greater than 5.5 reading was confirmed. We assumed that the pH test had the same sensitivities in the both scenarios. We tested the cost impact under the two scenarios given cut-offs 4‑6 (Additional files).

### Usability assessment

To understand experiences using the novel ester impregnated strips, we carried out a post-study survey of the 10 study sites. We solicited experiences of the research staff in NG-tube feeding, inquired about the patient number, assessed relative opinions of 1- and 2-min assay waiting times as well as ease of use of (1 extremely easy to 10 extremely difficult) and user confidence in the novel strip (1 not confident at all to 10 extremely confident).

### Adoption study

To map out potential route to adoption for the novel pH test, we hosted a workshop at the St Mary’s Hospital, London, on the 8 September 2017. The workshop was chaired by an expert in technology innovation from the Imperial College business school and attended by the research team at Imperial, representatives from the manufacturer and from the Oxford and Imperial Academic Health Science Networks (AHSNs). The research team presented the preliminary findings from the clinical study, followed by a round-table discussion of how the new strips could be brought into the NHS. In order to assess the potential need for the novel pH test at international level, we launched an international survey of clinical practice and the opinions of using pH tests and X-rays by clinical specialists, recruited through an author’s memberships (FR) in a range of international societies (Additional files).

### Outcome measures

The primary end point is the accuracy of novel and standard pH tests at the recommended pH cut-off of 5.5. Secondary end points are (i) the accuracy of novel and standard pH tests at other pH cut-off values of 4.0, 5.0 and 6.0; (ii) reliability of reading pH tests; (iii) radiography cost savings to the NHS and (iv) usability of novel pH test.

### Statistical analyses

Based on the results from the pilot study [19], and assuming that the novel test has a sensitivity of 90% and the standard pH test has a sensitivity of 70%, we set the recruitment target at 145 stomach samples in order to achieve significant statistical differences with power of 80% and the level of significance at 5%, taking into account loss from incomplete or unsuitable samples (10%) based on a two-sample proportion test. We considered all the data collected throughout the entire study period. Patients who could not be aspirated were excluded, as were samples with readings from only one strip, either of the standard or of the novel strips. When the readings from both readers existed, we used their averages, rounded to one decimal place, as the final pH reading.

Diagnostic performance was assessed in terms of the sensitivity of the pH test towards stomach placements. Test sensitivity was defined as the proportion of pH readings of the gastric placements that were equal to or below cut-off values of 4.0, 5.0, 5.5 and 6.0, which are commonly used in practice [15]. We reported both the mean values and the 95% confidence intervals at each pH cut-off. For gastric samples, the difference between the mean pH readings under the two strips was assessed using paired *t* tests and the difference between test sensitivities was assessed using McNemar’s Chi-squared test for paired samples. Inter-rater reliability was tested by Kappa statistics. We assessed variability in the test sensitivity across 10 study sites using a funnel plot (Additional files). We also reported the mean and confidence interval of the pH of the lung samples which we collected from the patients being intubated for cancer surgeries at the St Mary’s hospital.

All the data analyses were performed using the open source statistical software *R* for Mac OS X (version 3.5.0). A significance level of 0.05 was adopted and all tests were two sided.

All authors had access to the study data and reviewed and approved the final manuscript.

### Patient and public involvement

Patients or the public were not involved in the design, or conduct, or reporting, or dissemination plans of our research.

## Results

### Quality control of ester impregnated pH strips

A total of 6400 tributyrin-impregnated pH strips, in batches of 400 strips, were provided for the clinical study. We sampled from each manufactured batch and tested for their response to solutions containing lipase or the water control showed consistent results. All modified pH strips exposed to the water control typically read pH 7 (+/−0.5 units), modified strips exposed to PPL typically read pH 4.0 (+/−0.5 units) and modified strips exposed to CAL typically read pH 3.5 (+/−0.5 units) (Table 1). Unmodified pH strips typically read pH 7.0 (+/−0.5 units) when exposed to all 3 solutions. The required specification for each batch of modified pH strips released for use in the clinical diagnostic study was that all 5 strips exposed to either lipase read equal or less than pH 5.0. No change in modified pH strip response to either lipase was observed after 12 months storage in standard packaging at ambient temperature.

**Table 1:** Replicates of pH readings from control (standard Enteral) strips and tributyrin impregnated strips exposed to solutions of water (W), 100 U/ml porcine pancreatic lipase (PPL) and 100 U/ml *Candida antarctica* lipase (CAL)

	Replicate 1			Replicate 2			Replicate 3			Replicate 4			Replicate 5		
Solutions applied to pH strips	W	PPL	CAL	W	PPL	CAL	W	PPL	CAL	W	PPL	CAL	W	PPL	CAL
pH readings: control strip	7	4	3.5	7	4	3.5	7	4	3.5	7	4	3.5	7	4	3.5
pH readings: tributyrin strip	7	7	7	7	7	7	7	7	7	7	7	7	7	7	7

### Diagnostic accuracy study

**Fig. 1:**
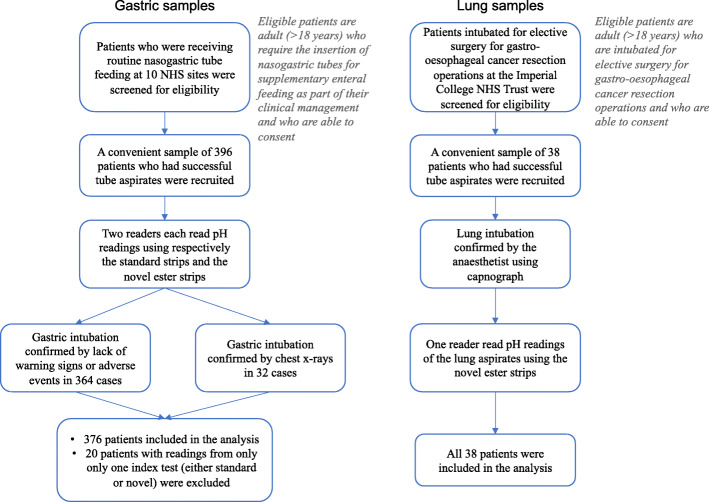
Study flow chart

A total number of 396 patients were recruited between January 2017 and April 2018 from 10 acute NHS trusts in England. Of these, we removed 20 patients who had readings from only one index test (either standard or novel), leaving the final sample size at 376 (Fig. 1). The mean age of the patients was 65.2 ± 14.7 (range 19‑95) with an average BMI of 25.8 ± 6.3 (range 14‑55). The male/female ratio was 60/40. Of 376 patients, 330 (or 88%) patients were on acid suppression medication and 316 (84%) had not been fasting prior to the pH testing.

**Fig. 2:**
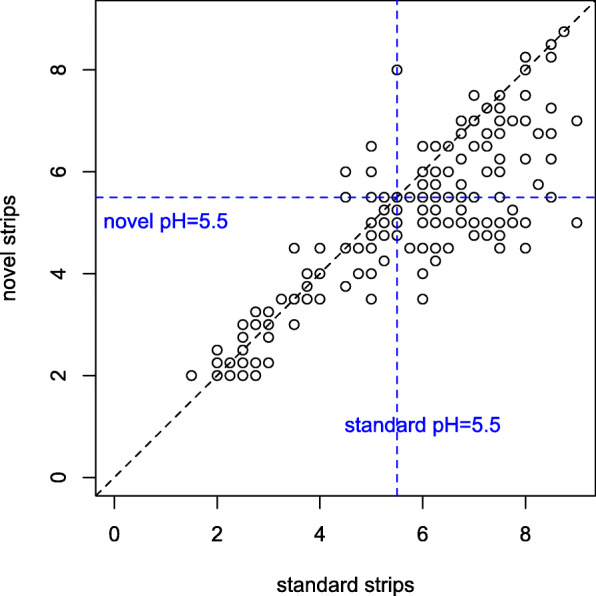
Gastric pH of 376 patients tested under standard strips (*x*-axis) and novel ester-impregnated strips (*y*-axis). Each dot corresponds to one data point. Horizontal and vertical lines indicate the recommended pH cut-offs of 5.5. The diagonal line indicated where the pH readings were identical under the novel and standard strips. All the dots beneath (above) the diagonal lines indicated those pH readings that were higher (lower) under the standard strips than under the novel strips

**Fig. 3:**
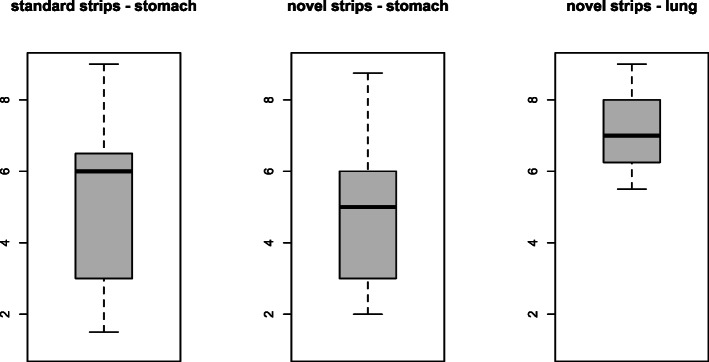
Boxplots comparing pH of gastric samples using the standard strips (left) and the ester-impregnated strips (middle) and pH of lung samples (retrieved during surgery) using the ester-impregnated strips (right)

All patients had tubes inserted inside the stomach confirmed by either CXR or in its absence, clinical observations. No adverse feeding incidents had been reported. Under the recommended cut-off of pH 5.5, the sensitivity of the standard test was 49.2% (185/376, 95% CI 44.1%, 54.3%) and the sensitivity of the novel test was 70.2% (264/376, 95% CI 65.6%, 74.8%) observed in the 376 patients. In other words, the novel test had a margin of 21% (95% CI 14.2‑27.9%) (=70.2‑49.2%) against the standard test. Of the 376 patients with the valid pH readings, 169 (45.9%) had pH readings that were lower under the novel strips than under the standard strips, 171 (45.5%) had identical readings and 36 (9.6%) had pH readings that were higher under the novel strips (Fig. 2). Overall the pH from the novel strips were significantly lower than the standard strips (mean 4.62 vs. 5.06, *p* = 0.002, Fig. 3). The novel test was more sensitive than the standard Enteral test under all cut-offs except for cut-off 4, where the two tests showed similar sensitivities (Table 2). The inter-rater reliability for the novel and standard strips did not differ (kappa scores for novel 0.773 and for standard 0.766).

**Table 2:** Sensitivities (95% CI) of the standard (Enteral) and the novel ester impregnated pH strips (*n* = 376) under pH cut-off 4, 5, 5.5 and 6

Cut-off	% pH ≤ cut-off under standard strips	% pH ≤ cut-off under novel strips	Difference (novel-standard)	*p* value of difference^a^
4.0	33.2% (125/376) (28.4%, 38.0%)	35.6% (134/376) (30.8%, 40.4%)	2.4% (−4.4%, 9.2%)	0.03
5.0	43.4% (163/376) (38.4%, 48.4%)	56.1% (211/376) (51.1%, 61.1%)	12.7% (5.6%, 19.8%)	< 0.0001
5.5	49.2% (185/376) (44.1%, 54.3%)	70.2% (264/376) (65.6%, 74.8%)	21% (14.2%, 27.8%)	< 0.0001
6.0	64.6% (243/376) (59.8%, 69.4%)	83.0% (312/376) (79.2%, 86.8%)	18.4% (12.3%, 24.5%)	< 0.0001

^a^McNemar’s Chi-squared test for paired samples

We also collected 38 lung samples from intubated patients whilst in theatre receiving elective surgery for gastro-oesophageal cancer resections. These were tested on the ester impregnated strips, which showed that the mean lung pH was 7.15 (95% CI 6.78, 7.52). None of the 38 samples were below pH 5.5, although four had the borderline reading of pH 5.5. The lung specificities of the novel strips were 100% at cut-off 5 or below, 89.5% (34/38, 95% CI 79.6%, 99.4%) at cut-off 5.5 and 78.9% (30/38, 95% CI, 65.8%, 92.1%) at cut-off 6.

### Cost-saving assessment

To understand the potential cost savings due to a reduction in CXRs, we considered 1000 hypothetical eligible patients assuming that that the novel and standard strips would cost the same. Based on our assumption, 900 of these patients (90%) had tubes blindly inserted inside the stomach and successful aspiration was achieved in 630 patients (70% at first attempt). Of these, the standard strips which had a sensitivity of 49.2% would correctly identify 310 patients (= 630 × 49.20%) as having a pH at or below 5.5, compared to 442 patients (= 630 × 70.20%) identified using the novel ester impregnated strips which had a sensitivity of 70.2%. Under the current guideline, CXRs are recommended for patients with a pH greater than 5.5, or 320 (= 630‑310) patients if the standard strips were used and 188 (= 630‑442) if the novel strips were used. Our assumptions are that chest X-rays would have been used on all 270 patients since aspirates are not available. Therefore, the novel strip could potentially save 132 (=320‑188) unnecessary chest X-rays per 1000 eligible patient checks. This translates into direct monetary savings of £3766 (=£28.53×132), using the NHS reference price for CXRs of £28.53 per test without adjustment of inflation or £4034 per 1000 patient checks, when inflation-adjusted .

The savings were sensitive to the accuracy of the pH strips. Of the 10 trial sites (Additional files), the novel strips outperformed the standard strips with a margin that varied from 5.6% at one site to 37.9% at another. Potential reduction of unnecessary chest X-rays thus varied from 35 to 239 and associated cost savings from £999 (= £28.53×35) to £6819 (= £28.53×239), per 1000 patient checks before inflation or between £1070 and £7304 when inflation-adjusted.

In the more conservative ‘recheck’ scenario, where we assumed that a second pH test was carried out on the sub-group of patients who had a pH higher than 5.5 at first attempt. In this scenario, the novel strip would still save 109 CXRs, equivalent to a saving £3110 (= £28.53×109), per 1000 eligible patient checks under cut-off of pH 5.5, or £3331 after adjusting for inflation. The savings were smaller at other pH cut-offs (4, 5, 6) where the difference in test sensitivity between the novel and the standard strips was smaller compared to that at pH 5.5 (Table 2).

### Usability and adoption

Post-study survey showed that the study teams had between 5 and 30 years of experience in nasogastric tube feeding. Successful aspirations were achieved 90% of the time (range 50‑100%). The clinicians found it relatively easy to wait for 2 min when using the novel lipase test (mean 2.78, out of 1 extremely easy—10 extremely difficult) instead of 1 min when using the standard strips. They were also confident using the novel strips (mean 8.44, range 5‑10, 1 not confident at all—10 extremely confident). The participants of the adoption workshop expressed interests in using the novel strips and had a strong preference for the novel strips with a design that could further reduce human errors in pH reading and interpretation. Such a preference would suggest a point-of-care device with binary (yes/no) results, indicating the presence or absence of human lipase found in the tube aspirates.

A total number of 178 clinicians responded, including 63% doctors, 29% nurses and 15% dieticians, the majority being from the UK (46.62%), followed by mainland Europe (13.56%) and India (12.5%). Just over half (54.8%) of the respondents worked in a teaching hospital, with over 500 beds and between 10 and 30 beds in the intensive care unit. The survey respondents noted the risks of radiation and delays associated with the use of CXRs, especially in children younger than 10 years (Additional files). Seventy percent of the participants expressed willingness to use the novel strips given NICE approvals.

## Discussion

We developed the first-generation ester-impregnated pH paper as a bedside test for locating blindly inserted NG-tubes in adult patients. The novel test achieved the desired analytical validity in laboratory quality control testing. In the clinical study with 376 patients receiving routine NG-tube feeding, the novel paper demonstrated significantly higher sensitivity than the standard test in confirming stomach placements under the commonly used pH cut-offs including 5.0, 5.5 and 6.0. The largest improvement in sensitivity was found under the recommended cut-off 5.5, whereby the novel strip detected 70.2% of the gastric samples compared to 49.2% using the standard strip, or a 21% difference (95% CI 14.2%, 27.8%). Under the current safety guideline, patients are sent for a confirmatory chest X-ray when an aspirate pH above 5.5 has been established. Therefore, using the ester impregnated strips could potentially save 132 CXRs per check of 1000 eligible patients, worth £4034 in CXR cost alone. Additional cost savings and efficiency gains could be achieved when we consider administrative burdens and hospital bed occupancy management associated with delay in CXR requests. Although the dual-marker novel pH strips prescribed a 2-min waiting time between strip impregnation and results, which is longer than the 1-min waiting time required by the standard strips, the clinicians found this acceptable.

The gastric pH tested by the standard strips in our studies was higher than reported in other studies [9, 15], and the sensitivity of the standard strips is considerably lower compared to a recent paper [9], which reported 68% (95% CI 57‑77%). However, their results were observed in samples obtained from patients undergoing gastroscopy. The patients in our study were real-life patients receiving feeding and medication, both could elevate gastric pH. Neither was fasting an inclusion criterion, which could give rise to hypochlorhydria, which then reduces human gastric lipase secretion [24]. This in turn reduces the extent to which an aspirate can reduce the baseline pH readings. This chain reaction could explain in part why a recent study failed to identify any differences in test sensitivity when the HGL-/tributyrin-based test from the same provider was compared against the standard pH test [25].

As expected, the lung pH measured by the novel strips (mean 7.15, 95% CI 6.78, 7.52) was significantly higher than the gastric pH measured by both standard strips (mean 5.06, 95% CI 4.86‑5.27) and the novel strips (mean 4.62, 95% CI 4.44‑4.80). Out of the 38 lung samples obtained during surgery, four samples had a pH reading of 5.5. We believe that this was due to micro-aspiration which occurs when secretions migrate from the oesophagus through an underinflated tracheal cuff or through longitudinal folds in high volume-low pressure cuffs of feeding tubes [26]. Micro-aspiration is common in patients undergoing surgery but unlikely to arise in ward patients, which is the target population for the pH testing. Previous testing of the tributyrin-impregnated strips on 99 aspirates retrieved from the lung under non-surgical conditions showed no readings ≤ pH 5.5 [25] and no evidence of lipase presence in the lung [19].

We had previously recommended reducing the recommended pH cut-off from 5.5 to 4, in order to lower the risks of feeding into the oesophagus [27]. Whilst a lower cut-off was considered safer, this recommendation was not implemented due to an anticipated increase in chest X-ray requests [5]. However, the novel strip of this study would have superior sensitivity under the cut-off of pH 5 (56.1%) than the standard strip under the cut-off of pH 5.5 (49.2%), or a margin of 6.9% (95% CI −0.2%, 14%). This suggests it would be possible to reduce both mis-feeding and the cost of confirmatory chest X-rays by using the novel strip under a lower safe-feeding cut-off of pH 5.

Our study has a number of limitations. Firstly, our primary aim was to test the hypothesis that the sensitivity of the novel strips would be higher compared to the standard strips. Instead of a single-gate study, we employed a two-gate design wherein we also collected lung aspirates. This is because one of the major safety concerns associated with NG feeding was feeding into the lung. However, since it was not possible to collect lung samples from feeding tubes inadvertently inserted into the lung, we instead collected from a different cohort of patients undergoing cancer surgeries. The insights generated will be used to refine test performance and guide its appropriate clinical applications. Secondly, we directly compared the novel strips to standard strips from a leading pH strip provider (Enteral). Although these strips are widely used in the UK and elsewhere, the performance of strips from different manufacturers may vary. Since the Enteral strips served as the base strip for manufacture of the ester impregnated strips, further study is required to assess the effect of ester impregnation upon strips from other providers. Additionally, since the readers of the novel strips were not blinded to the readings from the standard strips, the readings both between the two strips and between the two readers were likely correlated, a likely source of bias we will investigate in future studies. Thirdly, the sensitivity of the strips varied across the 10 trial sites. The largest difference between novel and standard strips was 37.9% (81.0% novel vs. 43.1% standard, *n* = 53) compared to the smallest difference which was 5.6% (55.6% novel vs. 50.0% standard, *n* = 18). Such variations cannot be accounted for by sample size alone (Additional files). We did not identify any learning effect in reading pH strips and the patients were of a similar profile across the 10 hospitals. We suspect underlying human factor issues that may explain the variation but confirming these would warrant future research. Lastly, more robust data in terms of the actual proportion of successful aspirations and of lung specificities of the novel test would strengthen the robustness of the health economic analysis.

## Conclusions

We have developed and validated a first-generation ester-impregnated pH strip test for locating nasogastric tubes prior to feeding in adult patients. The novel pH test was found to be 21% more sensitive than the standard pH test in 376 patients under the recommended cut-off of 5.5. Replacing standard pH strips with the novel pH strips could significantly reduce unnecessary chest X-rays and their associated costs. Using the more sensitive pH strips could support a reduction of the recommended safe feeding cut-off to pH ≤ 5.0 thereby enhancing patient safety whilst still reducing confirmatory X-rays.

## Supplementary Information

**Additional file 1.** Preparation and Quality Control testing of Tributyrin-impregnated pH strips. Preparation of ester impregnated pH strips. Quality Control protocol to test ester impregnated pH strips. Figure A1. Timeline of the multi-centre diagnostic performance study. Gastric study site recruitment, trouble shooting and results. Site recruitment and adoption. Trouble shooting and protocol amendment. Gastric study results by sites. Figure A2. Funnel plot with 95% control limits showing performance of standard strips (left) and novel strips (right) - appendix. Figure A3. Difference in sensitivity between novel and standard strips by site. Cost impact on the NHS. Methods. Results. Figure A4. Clinical pathways of nasogastric tube feeding under the standard scenario (left) and the recheck scenario (right). Table 2. Distribution of feeding outcomes in 1000 patients with 700 gastric placements in standard and recheck scenarios. Post-study survey questionnaire. On-line survey of international experts in NG-tube feeding. Aims and methods. Key results. On-line survey questions.

**Additional file 2.** A diagnostic accuracy study to evaluate point of Care lipase/pH test strip to confirm correct nasogastric position – Version 9

## Data Availability

Data are available upon reasonable requests.

## Abbreviations

NG-tubes: Nasogastric tubes
CXR: Chest X-rays
HGL: Human gastric lipase
HCl: Gastric hydrochloric acid
PPIs: Proton pump inhibitors
PPL: Porcine pancreatic lipase
CAL: Candida Antarctica lipase
CRN: Clinical Research Network
AHSNs: Academic Health Science Networks
