# Supplementary material for: Development and validation of ester impregnated pH strips for locating nasogastric feeding tubes in the stomach—a multicentre prospective diagnostic performance study
Source: Diagn Progn Res. 2021 Dec 14;5:22. doi: 10.1186/s41512-021-00111-9 (PMC8670038; doi:10.1186/s41512-021-00111-9)
Supplement: Supplementary file 1 — Additional file 1. Preparation and Quality Control testing of Tributyrin-impregnated pH strips. Preparation of ester impregnated pH strips. Quality Control protocol to test ester impregnated pH strips. Figure A1. Timeline of the multi-centre diagnostic performance study. Gastric study site recruitment, trouble shooting and results. Site recruitment and adoption. Trouble shooting and protocol amendment. Gastric study results by sites. Figure A2. Funnel plot with 95% control limits showing performance of standard strips (left) and novel strips (right) - appendix. Figure A3. Difference in sensitivity between novel and standard strips by site. Cost impact on the NHS. Methods. Results. Figure A4. Clinical pathways of nasogastric tube feeding under the standard scenario (left) and the recheck scenario (right). Table 2. Distribution of feeding outcomes in 1000 patients with 700 gastric placements in standard and recheck scenarios. Post-study survey questionnaire. On-line survey of international experts in NG-tube feeding. Aims and methods. Key results. On-line survey questions. [file 41512_2021_111_MOESM1_ESM.docx]

# ***Development and validation of ester impregnated pH strips for locating nasogastric feeding tubes in the stomach – a multicentre prospective diagnostic accuracy study***

*Melody Ni^1^, Mina E Adam^1^, Fatima Akbar^1^, Jeremy R Huddy^1^, Simone Borsci^1,2^, Peter Buckle^1^, Francesca Rubulotta^3^, Reuben Carr^4^, Ian Fotheringham^4^, Claire Wilson^4^, Matthew Tsang^4^_,_ Susan Harding^6^, Nicola White^7^ and George B Hanna^1^*

**Supplementary material**

[1. Preparation and Quality Control testing of Tributyrin-impregnated pH strips 2](#_Toc28147464)

[1.1 Preparation of ester impregnated pH strips 2](#_Toc28147465)

[1.2 Quality Control protocol to test ester impregnated pH strips 2](#_Toc28147466)

[2. Gastric study site recruitment, trouble shooting and results 3](#_Toc28147467)

[2.1 Site recruitment and adoption 3](#_Toc28147468)

[2.2 Trouble shooting and protocol amendment 4](#_Toc28147469)

[2.3 Gastric study results by sites 5](#_Toc28147470)

[3. Cost impact on the NHS 7](#_Toc28147471)

[3.1 Methods 7](#_Toc28147472)

[3.2 Results 9](#_Toc28147473)

[4. Post-study survey questionnaire 11](#_Toc28147474)

[5. On-line survey of international experts in NG-tube feeding 13](#_Toc28147475)

[5.1 Aims and methoods 13](#_Toc28147476)

[5.2 Key results: 14](#_Toc28147477)

[5.3 On-line survey questions 14](#_Toc28147478)

## Preparation and Quality Control testing of Tributyrin-impregnated pH strips

### 1.1 Preparation of ester impregnated pH strips

All chemical reagents were obtained from Sigma-Aldrich (Sigma Ltd, Poole, Dorset, UK). Commercial pH indicator strips (GBUK/Enteral) were obtained from (GBUK/Enteral Ltd, North Duffield Selby, North Yorkshire, UK). A solution of 0.1 g sodium taurodeoxycholate hydrate, 0.9 g sodium chloride and 1mL 99% tributyrin was prepared in 99 mL molecular biology grade water. The solution was maintained at pH 7 using a pH-stat method by addition of 50mM sodium hydroxide using a Titrando 902 (Metrohm, Herisau, Switzerland). Standard pH indicator strips (GBUK/Enteral) were submerged in the tributyrin solution for 3 seconds and dried overnight in a vacuum oven with vacuum ≤30 mbar (Haraeus, Hanau, Germany). Dried tributyrin impregnated strips were then stored in a 50 mL falcon tube at ambient temperature.

### 1.2 Quality Control protocol to test ester impregnated pH strips

A 1ml solution of Porcine Pancreatic lipase (Sigma Ltd, Poole, Dorset, UK) was prepared in molecular biology grade water (VWR Ltd, Lutterworth, Leicestershire) to a lipase concentration of 100 Units/mL. A 1ml solution of *Candida antarctica* lipase (Sigma Ltd, Poole, Dorset,UK) was prepared in molecular biology grade water to a lipase concentration of 100 Units/mL. These three solutions were then used to obtain a set of pH readings from a control strip and from a tributyrin modified strip. A 90 µL quantity of the Porcine Pancreatic lipase solution at pH 7.0 was used to fully wet the reactive area of a first tributyrin modified pH indicator strip. A 90 µL quantity of the *Candida antarctica* lipase solution at pH 7.0 was used to fully wet a second tributyrin modified pH indicator strip. A 90 µL quantity of molecular biology grade water at pH 7.0 was used to wet a third tributyrin modified pH indicator strip. Identical quantities of each solution were also used to saturate unmodified pH indicator strips. All tests were performed in replicates of 5. All strips were incubated at ambient temperature for 2 minutes. The pH readout arising from modified and unmodified strips was determined using the manufacturer’s supplied pH indicator chart and values recorded. Modified indicator strip batches in which all showed pH 5.0 or less following application of each lipase solution were approved for trial use. The stability of modified pH strip performance was monitored by performing the Quality Control (QC) test described 12 months following strip preparation. This showed no observable difference in response to lipase solution. A total of 6400 modified pH strips were manufactured, released using ISO13485 compliant Quality Control and supplied for the diagnostic accuracy study.

## Gastric study site recruitment, trouble shooting and results

### 2.1 Site recruitment and adoption

The study was planned as a single site study and commenced in January 2017 at the **Imperial** College NHS Trust (St Mary’s and Charing Cross hospitals, London). In April 2017, the study was adopted by the UK NIHR Clinical Research Network (CRN) to form part of the UK NIHR research study portfolio. This enabled the adoption of the study at nine additional NHS sites. To ensure appropriate sampling and data collection quality, each site received a half-day training from the research practitioner (FA) from Imperial who was also the study coordinator.

We recruited a total of 9 additional NHS sites. These were, in the order of adoption, The **Medway** Maritime NHS Foundation Trust, The **Royal Bournemouth** and Christchurch Hospitals NHS Foundation Trust, The Lancashire Teaching Hospitals NHS Trust (**Royal Preston**), The Wye Valley NHS Trust (**Hereford**), The **North Devon** Healthcare NHS Trust, The Central **Manchester** University Hospitals NHS Foundation Trust, The University **Southampton** Hospital, The **Hampshire** Hospital NHS Foundation Trust, The **Maidstone** and Tunbridge Wells NHS Trust. To participate, each site completed an Expression of Interest (EOI) form, which Imperial reviewed to confirm site research experience, research capacity and patient capacity. Also declared on the EOI were competing studies that might influence the recruitment for the proposed study. Once accepted, a recruitment target was agreed with the site, which was typically either 50 for larger sites or between 10 and 20 for smaller ones. UK Health Research Authority (HRA) approvals were sought at individual sites. To ensure appropriate sampling and data collection quality, each site received a half-day training from the research practitioner (FA) from Imperial who was also the study coordinator. **Figure A1** shows the timeline of the study.

**Figure A1. Timeline of the multi-centre diagnostic performance study**

### 2.2 Trouble shooting and protocol amendment

In August 2017, during one of our routine quality checks of sample collection, we discovered that at one participating site the pH readings made from the novel strips were consistently higher than the readings from the standard strips. This was unexpected since, in principle, the novel test chemistry can provide only a comparable or *lowered* relative pH reading, a hypothesis fully supported by the earlier pilot study and data collected at two other sites[^11^](#_ENREF_11). Tests at Imperial were consistent with those of the site, ruling out reading errors. Sample collection was temporarily suspended (Figure A1) while a thorough investigation was conducted by Ingenza scientists on the technical performance of the batches of standard and novel strips in question.

This investigation identified that the time taken to reach a final pH readout of standard pH strips could be influenced by the volume of aspirate applied and that a degree of batch to batch variability was observed in the temporal response of standard pH strips to changes in the volume of applied aspirate that were of identical pH. Such batch to batch variability is of little or no significance in standard practice. However, in a direct comparison of the assay strips in laboratory tests, aberrant results were observed that are consistent with the unexplained results observed at the trial site. This aberrant result was hypothesised as being due to small variations in colorimetric pH indicator dye content between lots of pH paper. If the batch of standard pH strips used as control in the study is not the same batch used to prepare the tributyrin impregnated pH strips then the applied aspirate volume might become a limiting factor in the response of the different batches of paper. Notably this relatively minor effect would only manifest itself in aberrant diagnostic readouts in instances where both low concentrations of HGL and HCl were present in the aspirate sample. Additionally, we considered the time of pH strip colour development and potential confusion of (identically appearing) standard and novel strips to be potentially compounding sources of error in assessing the relative performance of standard and novel pH strips derived from different batches.

Following this investigation, we amended the strip preparation and study protocols accordingly to eliminate all these potential sources of error. Firstly, novel strips were marked with an ‘I’ whereas standard strips were marked with an ‘O’ to prevent confusion between strips. Secondly, each study site was supplied with novel and standard (control) strips originating from a single batch of standard pH strips. All strips were encased in a kit in which the test protocol was clearly delineated and where the importance of applying adequate and equivalent volumes of aspirate to each strip and waiting the full 2 minutes before recording strip readings were emphasised. In October 2017, we obtained UK HRA approval for these study protocol amendments. Boxes containing the two index tests were distributed to all sites and the study was re-opened. Imperial (FA) followed up with individual sites to ensure the correct execution of the new protocol. Subsequent routine monitoring showed no further abnormal results.

### 2.3 Gastric study results by sites

Across 10 different study sites, the diagnostic performance of the novel and the standard pH tests varied. Although this variation was largely driven by sample size, especially in standard strips, we observed unusually high and low sensitivities from using the novel strips in site B and F respectively (**Figure A2, right panel**). The relative performance of the novel strips versus the standard strips also varied, although not statistically significant (**Figure A3**). The largest difference found at Hospital A (n=58), where the gap between the novel and standard pH tests was 37.9% (novel/standard: 81.0% versus 43.1%) closely followed by Hospital B (n=53) with a gap of 35.8% between the two index tests (88.7% versus 52.8%, p<0.001 in both cases). The smallest difference was found at Hospital I (n=18) where the gap was 5.6% (95% CI: -27% - 38%, p>0.05).


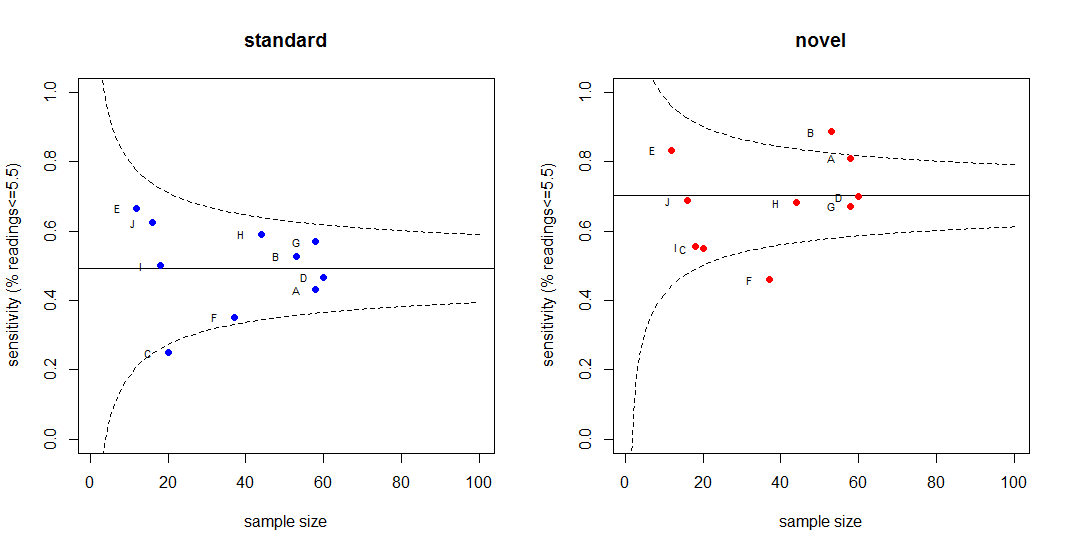


**Figure A2. Funnel plot with 95% control limits showing performance of standard strips (left) and novel strips (right) - appendix**


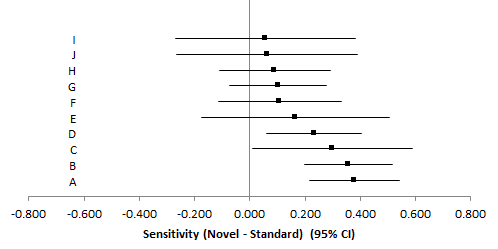


**Figure A3. Difference in sensitivity between novel and standard strips by site**

## Cost impact on the NHS

### 3.1 Methods

Using the sensitivity data from the diagnostic performance study, we estimated potential cost savings to the UK NHS, assuming that the novel acid/HGL based test strips would replace the standard pH strips as the first-line test for siting NG tubes. The clinical pathway was mapped out based on the current guideline (**Figure A4**). Under the standard scenario, chest x-rays are requested when a pH reading greater than 5.5 has been confirmed. Therefore, the more sensitive a pH test is, the fewer CXR requests are made and vice versa. In addition to this standard scenario (left panel, Figure 3), we considered a *recheck* scenario where instead of requesting chest x-rays as soon as a pH>5.5 reading was observed, a further pH test was carried out, and chest x-rays were only requested when a second pH>5.5 reading was confirmed (right panel, Figure 3). We assumed that the pH test had the same sensitivity in the standard single pH test scenario and the recheck double pH tests scenario.

We considered a hypothetical cohort of 1,000 eligible patients of whom 90% had tubes placed inside the stomach (i.e. 900). We assumed that the aspiration was successful 70% of the time in the first attempt and 90% in recheck. Direct costs of chest x-rays were based on UK NHS reference price. Cost of the novel strips was assumed to equal that of the standard strip and was therefore not analysed. We did not consider any costs of training or implementation since the novel strip had identical design and usability as the standard strips. Our analysis did not identify any effect of learning when using either strip. In sensitivity analyses, we assessed the impact on potential cost savings when a larger or smaller number of patients had tubes correctly inserted into the stomach (50%-90%) as well as when aspirations were less successful at first attempt (50%, 70%).

| **Standard (without recheck)** | **Recheck** |
| --- | --- |
| 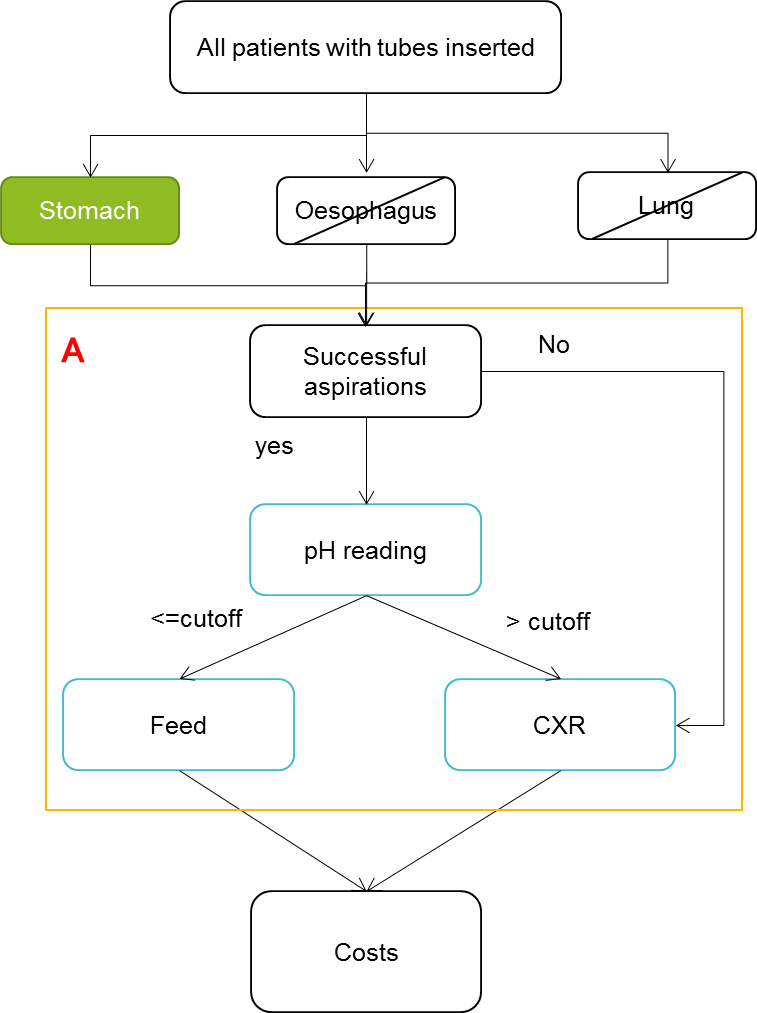 |  |
|  | 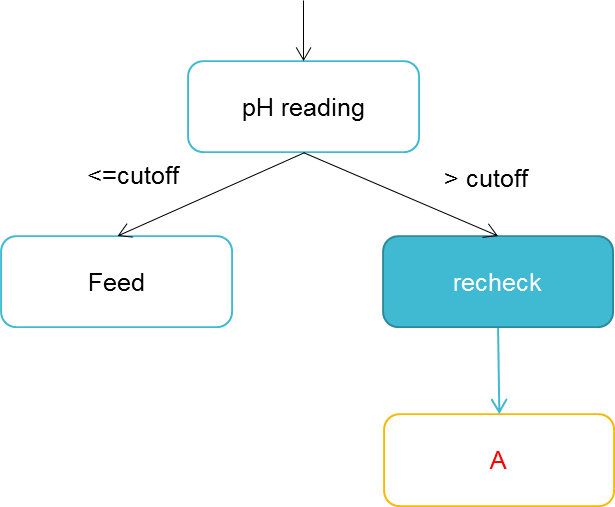 |

**Figure A4. Clinical pathways of nasogastric tube feeding under the standard scenario (left) and the recheck scenario (right)**

### 3.2 Results

Of the 1,000 hypothetical eligible patients, we assumed that 900 patients (90%) had tubes blindly inserted inside the stomach, of which successful aspiration was achieved in 630 patients (i.e. 70% at first attempt). **Table 2** showed the outcomes when the respective pH tests were used to confirm stomach placements in these 630 patients, using a pH reading that is equal to or less than the cut-off value. Considering a cut-off pH 5.5, using the novel test, 442 patients (~0.702*630) would test positive whereas using the standard strip, this number is 310 patients (~0.492*630). Therefore 132 (=442-310) unnecessary x-rays were saved using the novel strip instead of the standard strip to confirm gastric placements. Using the UK NHS reference price for chest x-ray (including labour) of £28.53 per test^[[1]](#footnote-1)^, this translates into a potential monetary savings in x-ray costs of £3,766 (=£28.53*132) per check per 1000 eligible patients without adjustment of inflation or £4,152 per 1000 patient checks, when inflation-adjusted (assumed at 5% p.a.). Across all other cutoffs, the novel test was also more sensitive than the standard test (**Table 1**). Therefore using the novel test would save unnecessary CXRs by between 15 (cut-off pH 4.0) and 132 (cut-off pH 5.5), corresponding to a potential monetary savings of between £428 (=£28.53*15) and £3,766, or £472 and £4,152 inflation-adjusted.

In the recheck scenario, where a second pH test was carried out prior to the chest x-ray, the novel strip would save unnecessary CXRs of between 19 (using cut-off pH 4.0) and 109 (using cut-off pH 5.5), corresponding to a potential monetary savings between £542 (=£28.53*19) and £3,110 (=£28.53*109) per check per 1000 eligible patients, or £598 and £4,152 inflation-adjusted.

.

**Table 2. Distribution of feeding outcomes in 1000 patients with 700 gastric placements in standard and recheck scenarios.**

| Cut-off | STANDARD (NO RECHECK) | | | | | RECHECK | | | | |
| --- | --- | --- | --- | --- | --- | --- | --- | --- | --- | --- |
|  | NOVEL   Strip | | STD  strip | | diff in CXR | NOVEL   Strip | | STD  strip | | diff in CXR |
|  | feed | CXR | feed | CXR | std-new | feed | CXR | feed | CXR | std-new |
| 4 | 224 | 406 | 209 | 421 | 15 | 354 | 276 | 335 | 295 | 19 |
| 5 | 353 | 277 | 273 | 357 | 80 | 493 | 137 | 413 | 217 | 80 |
| 5.5 | 442 | 188 | 310 | 320 | 132 | 561 | 69 | 452 | 178 | 109 |
| 6 | 523 | 107 | 407 | 223 | 116 | 603 | 27 | 537 | 93 | 66 |

Under sensitivity analyses, if 90% instead of 70% of the patients had successful aspirations after tube insertion, more patients would be eligible for the pH test and more patients would potentially benefit from the more sensitive novel strips. Using the novel strip could save as many as 170 unnecessary x-rays or a potential cost saving of £4,850 (=£28.53*170) per 1000 patients under the existing cut-off of pH 5.5, or £5,347 when inflation-adjusted. The worst-case scenario occurred when only 50% of the patients had tubes inserted inside the stomach, and successful aspirations were achieved in only half of them (50%). Only 250 patients out of the original 1000 would have tubes inside the stomach *and* provide aspirates to be tested using the pH strips. The novel test could still save 53 and 43 unnecessary x-rays under the standard and recheck scenarios, with a potential monetary cost savings of £1,512 (=£28.53*53) and £1,227 (=£28.53*43) respectively, when the cut-off of pH 5.5 was applied, or £1,667 and £1,352 when inflation-adjusted .

## Post-study survey questionnaire

The main research team at Imperial distributed the attached questionnaire to 10 study sites after the completion of study in April 2018. The main aim is to understand experiences in using the strips, and to identify any issues that might have emerged. We also solicited information in research nurse experiences and site capacity for NG-tube feeding. The survey showed that the study teams had between 5 and 30 years of experience in nasogastric tube feeding. Successful aspirations were achieved 90% of the time (range 50%-100%). The clinicians found it fairly easy to wait for 2 minutes when using the novel lipase test (mean 2.78, out of 1 extremely easy - 10 extremely difficult). They were also confident using the novel strips (mean 8.44, range 5-10, 1 not confident at all -10 extremely confident).

***A diagnostic study of a Point of Care Lipase pH test strip to confirm the correct nasogastric tube position***

**Principal Investigator:** Professor George Hanna, Professor of Surgical Sciences / Consultant Surgeon, Imperial College, St Mary’s Hospital, London

Site Name: ________________________

1. Please provide the names of specialty wards where the potentially suitable participants were screened from and consented in to the trial. Example; stroke ward, GI surgical ward, ICU etc.
2. List the main presenting symptoms or diagnosis that the eligible participants presented with?
3. Outline the sampling method used at your site. For example: were patients recruited on a consecutive basis from a particular ward?
4. How many months/years of experience did the research nurses have who were involved in reading the index test and reference standards?
5. Were there any adverse events recorded and reported from performing the index tests (i.e. novel pH strips) or the reference standard (i.e. chest x-rays)? If yes, please provide the details of these adverse events for all patients.
6. Please explain how the missing results were reported. For example, if there was a time when only one nurse was available to record the index test readings, what measures were applied to ensure missing data was reported.
7. How often were that tube aspirates cannot be successfully obtained and chest x-rays have to be used?
8. What is the approximate number of patients eligible for tube feeding in your hospital?
9. How easy or difficult do you find waiting for two minutes for the colour to change when using the novel strips compared to the standard strip? (1- extremely easy, 5 – neither easy nor difficult, 10 – extremely difficult)
10. How confident are you using the novel strip in actual clinical practice compared to the standard strip? (1- not confident at all, 5 – neutral, 10 – extremely confident)
11. Please tell us any difficulties you experienced using the novel strips.

*Thank you. We really appreciate you time*

## On-line survey of international experts in NG-tube feeding

### 5.1 Aims and methoods

Aim To understand variations in clinical practice in different countries for ensuring safety using blindly inserted NG-tubes to provide feeding and medication and to assess the market potential of the novel ester impregnated strips.

Design The research team (FR, SB and MN) developed the English language survey and distributed online between August 28^th^ and November 20^th^, 2017. The total number of questions was 39, constructed to be as easy to understand as possible. The questions were divided into demographics, currently practices used in ensuring safety in NG tube feeding, and the interest of using a more sensitive pH test instead of the current standard strips. Participants were given a description of the pH test based on its underlying mechanism (see Methods in the main test) and the design was a yes/no test that would further simplify the decision making process of feeding versus no-feeding. The language was tuned to suit non-native English speakers. The time to complete the questionnaire was controlled at approximately 10 minutes to maximise participation.

*Target audience* Doctors and nurses working in ICU or those who are familiar with the use of enteral feeding)

*Pilot* The draft questionnaire was piloted in 30 volunteers, with input from non-native English speakers. Clarification of responses was sought by e-mail and telephone.

*Data analysis* The survey was embedded in the Qualtrics platform hosted at Imperial College London. The platform supported data collection and basic data analysis. We reported relevant responses based on the analysis automatically generated by Qualtrics.

*Distribution* The survey link generated by the Qualtrics was accompanied by an cover letter which explained the purpose of the survey. The questionnaire was distributed via learned socities in Europe and internationally (see below), as well as on social media, e.g. Linkedin, twitter. Reminders were sent to those that had started and not completed the questionnaire (the aim was 100% response rate for the e mail distribution). After the survey, a thank-you letter and brief description of the results were distributed. The following professional organisations which helped the distribution of the survey were:

- The European Society of Intensive Care Medicine (ESICM)
- The he European Society for Clinical Nutrition and Metabolism (ESPEN)
- The European Competence Based Training in Intensive Care Medicine
- The Intensive Care Society (ICS) in the UK
- The Austrian Intensive Care Society
- The Polish Society of Anaesthesiology
- The Portuguese Intensive Care Society
- The Irish Society of Intensive Care
- Italian Society (SIAARTI)
- The Emirates Critical Care Congress (ECCC) and
- The South African Ventilation through the ages group (VTA).
- The Indian ESICM

### 5.2 Key results:

Of the 335 clinical experts invited, 178 responded (63% doctors, 29% nurses and 15% dieticians) who were from the UK (46.62%), India (12.5%), Europe (13.56%), USA/Canada (2.7%) and South Africa (1.35%). 54.8% of the respondents worked in a teaching hospital, and 10% of those in a private hospital. 60% of those had over 500 beds and 50% of those had between 10-30 beds in the intensive care unit.

90% of the participants confirmed that policies are in place which recommended the use of either or a combination of the following methods: pH strips, chest x-rays and other methods (e.g. visual inspection). 36.5% of the respondents used only CXRs as the checking methods. When asked about the pH cutoffs, 80.69% of the parcipants used a cut-off of 5.5 as indication of feeding. 43% of the time, tube aspirates cannot be obtained from the NG tube. However, 79% of the respondents replied that the patients would receive between 1 and 5 x-rays every 30 days. 48% of the respondents check tube locations on a daily basis, whilst 26% carried out checks only if they were worried or a colleague expressed concerns.suspected misplacements were most commonly checked by CXR (39.8%), and less so by visual inspection (23%) or pH strips followed by CXR (27%)

Respondents considered radiology exposure had a moderate level of risks to patients (2.6 out of 0-5, with higher score being higher risks), which was however more pronounced in children younger than 10 years old (60.5 out of the maximum 100). 58% of the respondents believed it took between 30 and 60 minutes to obtain an x-ray although in 7% of the cases, it can take 5 hours. Based on the description given in terms of the novel ester impregnated binary pH test, the participants were moderately confident (5.2 out of a maximum score of 10) about its clinical use although 70% of the participants said they would use it should NICE approved its use.

### 5.3 On-line survey questions

Q1-6: demographics

Q7 - How many beds are there in your hospital?

Q8 - How many beds are there in your Intensive Care Unit?

Q9 - Do you have any policies (National Guidelines, hospital policies or written instructions) to guide you in confirming the position of the NG tube before feeding a patient?

Q10 - Please specify which method is indicated in your policies to ascertain the position of NG tubes:

Q11 - How likely are your colleagues to be compliant when your hospital's policy suggests using only a chest X-ray to ascertain the NG Tube position?

Q12 - When using pH strips (alone or in combination), is a pH equal or below 5.5 indicated as a cut off to decide if it is safe to start feeding a patient?

Q13 - When you use pH strips for NG tube confirmation, how likely are you to request a chest X-ray because of uncertain results from the pH test?

Q14 - How often do you have no aspirate from the NG tube?

Q15 - Do you use pH strips as a method to ascertain that NG tube is in the stomach?

Q16 - In your current practice, which is the likelihood that one of the following methods is used to ascertain that the NG tube is in the stomach?

Q17 - When using pH strips (alone or in combination), is a pH equal or below 5.5 indicated as a cut off to decide if it is safe to start feeding a patient?

Q18 - When you use pH strips, how likely are you to request a chest X-ray because of uncertain results from the pH test?

Q19 - When you use pH strips how often do you have no aspirate from the NG tube?

Q20 - How often do you check if the NG tube is in the right place in order to avoid a misplacement?

Q21 - Which methods do you use to check the NG tube following the initial confirmation?

Q22 - Which methods do you use to check a suspected misplacement of the NG tube before feeding? (select as many as applicable)

Q23 - In your experience, after the insertion of the NG tube, how often does a patient need a chest X-ray to confirm that NG tube is still in the stomach?

Q24 - In your experience, how often does a chest X ray confirm that the NG tube is positioned outside the stomach?

Q25 - On average, after how many weeks of NG enteral nutrition would you consider alternative approaches for long term feeding e.g. Endoscopic Gastrostomy (PEG), or Radiologically Inserted Gastrostomy (RIG)?

Q26 - What are most common clinical causes or conditions that require prolonged enteral feeding in your hospital or ward?

Q29 - How many chest X rays does a patient who requires enteral feeding in your unit would receive for the only purpose of confirming the position of the NG tube during a 30 days hospitalisation?

Q30 - Considering the scenario we presented above, how would you rate, from 0 (Very low) to 5 (Very high), the total radiological risk (i.e., not only chest X-ray) for this critically ill patient after 30 days?

Q31 - Can you score the radiological risk for each of the following age groups knowing that there is a clear age vulnerability and variability reported in the literature?

Q32 - To the best of your knowledge, how much is the average cost for a chest X-ray in your institution?

Q33 - To the best of your knowledge, how long is the overall time needed from the request to the report of a chest X-ray results at your institution?

Q36 - Have you been formally trained in using pH strips in your institution?

Q37 - How much would you rate, from 0 (Very low) to 10 (Very high), your confidence in correctly ascertaining the position of the NG tube using only the new strips?

Q38 - On the basis of the description above, how likely would you be to use this new pH strips if quality control bodies or NICE will indicate they are the gold standard?

Q39 - On the basis of the description above, how much would you be willing to pay if this bi functional pH strips would be accepted as the gold standard? Please, assume that the average cost to buy one mono functional strip is equal to Euro 10 cent ($11.9 penny). However the package contains 100 strips so you must buy it for 10 euros ($11.9)

1. Reference Cost Collection: National Schedule of Reference Costs, 2016-17 - NHS trusts and NHS foundation trusts - Subcontracted data, NHS Improvement [↑](#footnote-ref-1)
