# Supplementary material for: Development and validation of ester impregnated pH strips for locating nasogastric feeding tubes in the stomach—a multicentre prospective diagnostic performance study
Source: Diagn Progn Res. 2021 Dec 14;5:22. doi: 10.1186/s41512-021-00111-9 (PMC8670038; doi:10.1186/s41512-021-00111-9)
Supplement: Supplementary file 2 — Additional file 2. A diagnostic accuracy study to evaluate point of Care lipase/pH test strip to confirm correct nasogastric position – Version 9 [file 41512_2021_111_MOESM2_ESM.docx]

**A diagnostic accuracy study to evaluate point of Care lipase/pH test strip to confirm correct nasogastric position – Version 9**

**Principal Investigator:**

Professor George Hanna PhD FRCS

Professor of Surgical Sciences / Consultant Surgeon

Imperial College, St Mary’s Hospital, London

**Introduction**

At least 1 million nasogastric (NG) tubes are used in the UK each year, often for supplementary feeding [1]. They allow liquid feed to be passed directly into the stomach without requiring the patient to swallow. The National Patient Safety Agency issued guidelines recommending aspirate pH is tested before every feed and at least once every day to check nasogastric tube position and prevent harm from feeding into the lungs through a misplaced nasogastric tube.

Current best practice uses pH testing to ensure the tube is correctly positioned in the stomach. An acidic result (pH equal to or less than 5.5) indicates that the nasogastric tube is correctly positioned in the stomach and feeding is safe. If the result is not acidic (pH >5.5), a chest radiograph is indicated to check that the nasogastric tube is positioned in the stomach and not in the lungs [2]. However, up to 42% of hospital inpatients receive antacid medications that render the results of pH test paper falsely negative [3].

The ideal solution would be a test that was accurate despite non-acidic gastric aspirates, safe, point-of-care, and non-ionising. Human gastric lipase is an endogenous gastric enzyme, which starts the digestion of dietary triglyceride in the human stomach [4]. Chief cells secrete human gastric lipase entirely from the gastric fundus [5,6]. Human gastric lipase is relatively stable and its production, unlike the secretion of hydrochloric acid from gastric parietal cells, is not affected by antacid medications [7,8]. One barrier to a single reagent test is that human gastric lipase is inactivated by acidic stomach contents and therefore is unsuitable as a means of determining nasogastric tube position on its own. It has been suggested that a combined test for pH incorporating a gastric enzyme may be significantly more accurate than each in isolation [9]. The objective of this study is to validate a nasogastric tube position test that was compatible with non-acidic gastric aspirates by utilising human gastric lipase to lower the pH of gastric aspirates on pH test paper.

**Study aims**

- To determine the sensitivity and specificity of the lipase/pH test strip
- To determine how many chest radiographs would be avoided if the lipase/pH test strip was used instead of the standard pH test
- To observe in a simulated environment the use of lipase based pH test strip and analyse the perceived usefulness of this test from professional perspective.

**Study design**

**1. Diagnostic Accuracy Study**

*Primary outcome*

Sensitivity of the index test and the reference tests (standard pH test) under cut-off 5.5 with tube sites confirmed by either patient follow up or chest x-rays when applicable.

*Secondary outcomes*

1. Percentage unable to aspirate is measured by the number of patients for whom no aspirate is obtained out of the total number of patients for whom the attempts have been made before either successful aspiration or before chest x-rays has to be requested

2. Number of chest radiographs requested is recorded exactly as described, when aspirates and re-attempts both fail

*Subject selection and setting*

Participants will be recruited from Imperial College Healthcare NHS Trust at both the Charing Cross and St. Mary’s hospital sites. Eligible participants will be identified by the clinical team and recruited by a member of the research team. These will be patients that require nasogastric tube insertion as part of their clinical management such as patients following stroke, gastrointestinal surgery or with head and neck conditions. Therefore the study will be undertaken on the following wards: Charles-Pannett, intensive care, Charing Cross wards 9 west, 9 north, 10 south and 9 south. If it has been determined that a participant lacks the capacity to make a decision for himself, a personal consultee shall be consulted on behalf of the participant. In the event that a personal consultee is not available, a Nominated Consultee shall be consulted to make a decision whether the patient may be a participant in the study.

*Inclusion Criteria*

Patients who require the insertion of nasogastric tubes for supplementary enteral feeding as part of their clinical management

*Exclusion Criteria*

- Patients under the age of 18 years
- Prisoners
- Patients sectioned under the Mental Health Act

*Protocol*

The clinical trial will be a diagnostic accuracy study of two diagnostic tests and will be performed in accordance with the STARD guidelines for diagnostic tests [11]. The index tests will be (i) the novel lipase/pH test strip and (ii) standard pH test strip. The reference test used for comparison will be chest x-ray or where this is not required ‘trial by use’ (any chest x-rays undertaken would be decided and authorised by the participant’s clinical team who are clearly separated from the research team. Undergoing a chest x-ray does not from part of screening for the study and is not a requirement of the study protocol).

Ingenza novel lipase/pH test strip

The test is based on detecting organic acid released from a chemical ester impregnated onto pH paper by the action of human gastric lipase that is stable in gastric juice and resistant towards antacid medications, as well as unlikely to migrate. Ingenza have impregnated enteral pH indicator strips that are in current clinical use with emulsion formulations protected by intellectual property as human gastric lipase substrates. Therefore the novel test strips will provide a positive result in the presence of an acidic environment (pH less than or equal to 5.5) or gastric lipase.

Index testing

The nasogastric tube will be aspirated to obtain a sample of gastric fluid. This will either be immediately following insertion or as part of daily position confirmation that is routinely performed prior to initiation of nasogastric feeding. The aspirate will be used by the healthcare professional, if required, as part of routine care and the remaining sample passed to a member of the research team. The sample will then be used to wet the two index test strips. Two members of the research team will independently assess the results of the test papers at two minutes. If two members of the research team are not available then one member will test the papers and take photographs of the test papers so that a second member of the team can verify the findings. These investigators will be blinded to the results of the reference standard. For standard pH strips a pH of ≤5.5 will indicate correct tube placement and >5.5 indicated incorrect nasogastric tube position. For lipase/pH strip test a positive result will be determined according to the manufacturers recommendations.

Reference Testing

The reference standard test will consist of chest radiography or trial by use if chest radiography is not indicated. Consultant radiologists who will be blinded to the index test results will interpret chest radiographs. Criteria for correct nasogastric tube position on the chest radiograph include a straight vertical course near the midline passing through the carina and not following a bronchus with the tip below the diaphragm on the same side as the gastric bubble.

Patients with a nasogastric tube in situ will be assessed with both the index and reference standard test. The reference standard pH test will be administered first (according to manufacturer’s instructions) followed immediately by the Ingenza enzymatic nasogastric tube position test. The same researcher will perform both tests. The pH test will be performed first as it is known from the pilot study to be the least reliable and so less likely to influence interpretation of the new test. The two tests will be read by the same healthcare worker to minimise inter-reader variability. The standard pH test will be deemed positive if the reader assesses the pH to be greater than 5.5 (a subjective assessment made by comparing the pH strip to a colour intensity chart). If either test is positive (suggesting that a tube is misplaced) then the participant’s clinical team will be informed so that they can confirm correct tube placement before use. Therefore, aside from the potential increase in patients requiring tube position verification by x-ray there will be no change to standard care policy. Patients will be followed up until they have had a gold-standard test to confirm the position of their nasogastric feeding tube. This will either be by x-ray or when x-ray is not indicated ‘trial by use’ of the nasogastric feeding tube.

*Statistics*

We aim to collect data from 145 patients. The sample size is computed based on test validity findings from our earlier trial at the St Mary’s hospital and has 80% power and 5% significance level and includes a 15% allowance for drop-out.

Diagnostic performance will be expressed through sensitivity with 95 % confidence Intervals (95 % CI). Demographic and clinical characteristics will be expressed as percentages for categorical data, and median with interquartile ranges (IQR) for continuous data due to their non-normal distribution.

**2. Study of test’s ability to predict lung placement**

In addition to this diagnostic accuracy study a separate analysis of the novel test will be undertaken to assess the accuracy of the test in the presence of lung aspirates. This study will be undertaken as NG placement in the lung is an extremely rare event and unlikely to occur in the population in study 1. However, lung placement is a major cause of morbidity and mortality in the use of NG tubes and therefore this study will ensure that the new test is reliable able to recognise lung intubation. Lung aspirates will be obtained from patients who are intubated for elective surgery. Correct tube placement will be confirmed with capnography and therefore it is reliable that aspirates from the tubes are from the lung. The aspirates will be tested with the novel test strips in the same method as for study 1 and used to determine sensitivity. 50 samples will be tested.

*Subject selection and setting*

Patients intubated for elective surgery

*Exclusion Criteria*

- Patients under the age of 18 years
- Prisoners
- Patients sectioned under the Mental Health Act

**Human factor analysis**

*Subject selection and setting*

25 participants will be recruited from Imperial Healthcare NHS Trusts. Due to the nature of the task involved in using the pH strips, professionals with visual impairments, such as colour blindness, will be excluded for the evaluation cohort. The sample size is in keeping with ISO 62366 (2008) guidelines to ascertain perceivable usability defects and qualitative analyses. Simulation tests will be performed at St. Mary’s hospital facilities.

*Inclusion Criteria*

Professionals with at least one year of experience in the use of pH strips to ascertain nasogastric feeding tube position.

*Exclusion Criteria*

- Visual impairments including colour blindness
- Patients under the age of 18 years
- Patients who are unable to sign consent.
- Patients who do not provide consent.

*Simulation Protocol*

During the simulation professionals will undertake a set of scenarios that involve interaction with lipase/pH test strip. Participants will be asked to simulate the procedure of NG tube positioning and the use of lipase/pH test strip. Time and error of performances will be tracked, and standardised scales will be used to assess their experience with the lipase/pH strip (see appendix).

**Consent**

Participants will be identified by the clinical team according to their eligibility on the research study. For patients who can consent, the research study will be fully explained to them. They will be given the patient information leaflet to read, what has been mentioned by the researcher. Consenting participants will be given enough time to think and decide to participate on the study. These participants will be requested to sign the consent prior obtaining any demographic questions. They can withdraw anytime freely from the study without giving reason and no prejudicial effect on their subsequent treatment.

For patients who have been determined to lack the capacity to consent for themselves through the Mental Capacity Act (2005), a Personal/Nominated Consultee will be consulted. The Personal/Nominated Consultee will be the one to decide if the patient would have no objection to taking part in the study, and therefore will be given a Consultee Information Leaflet. They will be given time to think and decide their actions. If they have deemed that the patient will have no objections to participate, they will then sign a Consultee Declaration Form. During the course of the study, if or when the patients gain back their capacity to consent, they will be consented by the researchers following the process of consent for consenting participants. They can withdraw anytime freely from the study without giving reason and no prejudicial effect on their subsequent treatment.

**Adverse Events**

The sample is to be collected from the nasogastric tube. Aspiration will be performed by a trained healthcare professional. Privacy and dignity will be ensured to the patient at all times during the study. As required samples are gastric aspirate that are collected as part of the patients usual clinical care we do not foresee any safety issues to arise from study participation. Results from the lipase/pH test strip will not be used to direct clinical care.

**Ethics**

Appropriate ethical approval will be sought from the xxx Research Ethics Committee. We have previously been granted ethical approval for both a pilot study of the clinical test prototype (REC ref 10/H0724/76) and for qualitative research into proactive risk assessment of hazards associated with NG tubes (REC ref 09/H0724/76).

**Indemnity**

Imperial College London holds negligent harm and non-negligent harm insurance policies that apply to this study.

**Confidentiality**

The Chief Investigator will preserve the confidentiality of participants taking part in the study and is registered under the Data Protection Act.

**Audits**

The study may be subject to inspection and audit by Imperial College London under their remit as sponsor and other regulatory bodies to ensure adherence to GCP and the NHS Research Governance Framework for Health and Social Care (2nd edition).

**Publication Policy**

The workshop outputs will be written up for peer-reviewed journals or submitted to academic conferences.

**Funding**

The funding for this body of work will be from Innovate UK and Ingenza. [Grant number]

**Study Details Contact**

For general queries, supply of study documentation, and collection of data, please contact:

Mina Adam

Email: m.adam15@imperial.ac.uk Telephone: 07493152315

Address: Division of Surgery, Imperial College London, 10^th^ Floor QEQM Building, St. Mary’s Hospital, London, W2 1NY.

References

[1] Coombes R. NHS safety agency issues guidance on nasogastric tubes. BMJ 2005;330:438–0. doi:10.1136/bmj.330.7489.438.

[2] NPSA. Patient safety resources n.d.

[3] Taylor SJ, Clemente R. Confirmation of nasogastric tube position by pH testing. J Hum Nutr Diet 2005;18:371–5. doi:10.1111/j.1365-277X.2005.00635.x.

[4] Cohen M, Morgan RG, Hofmann AF. Lipolytic activity of human gastric and duodenal juice against medium and long chain triglycerides. Gastroenterology 1971;60:1–15.

[5] Abrams CK, Hamosh M, Lee TC, Ansher AF, Collen MJ, Lewis JH, et al. Gastric lipase: localization in the human stomach. Gastroenterology 1988;95:1460–4.

[6] Moreau H, Laugier R, Gargouri Y, Ferrato F, Verger R. Human preduodenal lipase is entirely of gastric fundic origin. Gastroenterology 1988;95:1221–6.

[7] Renou C, Carrière F, Ville E, Grandval P, Joubert-Collin M, Laugier R. Effects of lansoprazole on human gastric lipase secretion and intragastric lipolysis in healthy human volunteers. Digestion 2001;63:207–13. doi:51891.

[8] Ville E, Carrière F, Renou C, Laugier R. Physiological study of pH stability and sensitivity to pepsin of human gastric lipase. Digestion 2002;65:73–81. doi:57708.

[9] Anderson O, Carr R, Harbinson M, Hanna GB. Development and validation of a lipase nasogastric tube position test. BMJ Open Gastroenterol 2016:e000064. doi:10.1136/bmjgast-2015-000064.

[10] Menon DK, Chatfield DA. A Guide for Critical Care Settings Mental Capacity Act 2005 Guidance for Critical Care n.d.

[11] Bossuyt PM. Towards Complete and Accurate Reporting of Studies of Diagnostic Accuracy: The STARD Initiative. Clin Chem 2003;49:1–6. doi:10.1373/49.1.1.

Appendix

1. **Information About You**

We would like to collect some information about you, and your expertise in the use of pH Strips to checking the position of the nasogastric feeding tube.

| 1. Your place of work… *(please, mark all that apply)*  - Primary care - GP surgery - Primary care - Social services - Secondary care - NHS Hospital Trust - Secondary care – other, please specify: ________________________ - Tertiary care |
| --- |
| 1. Level of experience… *(please, mark all that apply)*  - Doctor - Nurse - Paramedic - Pharmacist - Physiotherapist - Dietician - Other, please specify: _______________________________ |
| 1. Your gender is  - Male - Female - I prefer not to say |
| 1. Are you used to checking the position of the NG tube with pH strips? *(please, mark only one box)*  - Not at all. - Yes, but only when I have to. - Yes, on regular basis. |
| 1. Do you suffer from colour vision deficiency (colour blindness)? *(please, mark only one box)*  - No - Yes |
| 1. Have you received training in the use of pH strips and nasogastric feeding tubes?  - Not at all. - Yes, I received informal training at _____________________Year: - Yes, I received formal training at _______________________Year: |
| 1. How many years of experience do you have using pH strips to check NG tube positioning? _______ |
| 1. Your Age:  - 18-24 years old - 24-35 years old - 36-45 years old - 45-54 years old - >54 years old |

1. **How much trust do you have in the use of [***the new pH Strips***] to check the nasogastric feeding tube positioning?**

**Instructions**: For each of the following statements, mark one box that best describes your trust in the use of [new pH Strips] to check nasogastric tube position **from 1 (strongly disagree) to 7 (strongly agree)**

| - - 1. I am totally comfortable working with [new pH Strips]. | 1 | 2 | 3 | 4 | 5 | 6 | 7 |
| --- | --- | --- | --- | --- | --- | --- | --- |
| - - 1. I feel very good about how things go when I use [new pH Strips]. | 1 | 2 | 3 | 4 | 5 | 6 | 7 |
| - - 1. I always feel confident that the right things will happen when I use [new pH Strips]. | 1 | 2 | 3 | 4 | 5 | 6 | 7 |
| - - 1. It appears that things will be fine when I use [new pH Strips]. | 1 | 2 | 3 | 4 | 5 | 6 | 7 |
| - - 1. I believe that most [new pH Strips] are effective at what they are designed to do. | 1 | 2 | 3 | 4 | 5 | 6 | 7 |
| - - 1. A large majority of [new pH Strips] are excellent. | 1 | 2 | 3 | 4 | 5 | 6 | 7 |
| - - 1. I think most [new pH Strips] enable me to do what I need to do. | 1 | 2 | 3 | 4 | 5 | 6 | 7 |
| - - 1. My typical approach is to trust new [new pH Strips] that I have never used before until they prove to me that I shouldn’t trust them. | 1 | 2 | 3 | 4 | 5 | 6 | 7 |
| - - 1. I usually trust a pH strip outcome until it give me a reason not to trust it. | 1 | 2 | 3 | 4 | 5 | 6 | 7 |
| - - 1. I rely on alternative bedside methods (not x-ray) for confirmation of nasogastric tube placement in addition to aspirate pH determination | 1 | 2 | 3 | 4 | 5 | 6 | 7 |

*The above 10 statements are adapted from recent research on trust in technology. See for reference– Mcknight, D. H., et al. (2011). Trust in a specific technology: An investigation of its components and measures.*

1. **Perceived experience in use**

The following statements have been used by others to assess the perceived experience in the use of a tools.

In this case, we will ask to you to answer the questions in relation to the use of **current** **[***the new pH Strips***] to ascertain NG tube positioning**.

Please follow the instructions and fill in the questionnaire

**Instructions:** For each of the following statements, mark one box that best describes your overall experience with **current** **[new pH Strips] to ascertain NG tube positioning***:*

|  | Strongly Disagree |  |  |  |  |  | Strongly Agree |
| --- | --- | --- | --- | --- | --- | --- | --- |
| 1. [new pH Strips] capabilities meet my requirements | 1 | 2 | 3 | 4 | 5 | 6 | 7 |
| 1. [new pH Strips] are easy to use | 1 | 2 | 3 | 4 | 5 | 6 | 7 |

* See for reference – Finstad, K. (2010) The usability metric for user experience.

1. **Expectations**

By assuming the reliability of **[***the new pH Strips***]**, please rate your agreement with the following statements.

|  | Strongly Disagree |  |  |  |  |  | Strongly Agree |
| --- | --- | --- | --- | --- | --- | --- | --- |
| I believe that I will prefer to use an dual functioning gastric acid and [*the new pH Strips*] instead of a pH strip to ascertain the NG tube positioning | 1 | 2 | 3 | 4 | 5 | 6 | 7 |
| I believe that I will have a lowered need to use X-ray to confirm a NG tube positioning if I will have [*the new pH Strips*] | 1 | 2 | 3 | 4 | 5 | 6 | 7 |

1. **Your intention to use [***the new pH Strips***]***

***Instructions:*** mark one box that best describes your intention of use.

|  | Strongly Disagree |  |  |  |  |  | Strongly Agree |
| --- | --- | --- | --- | --- | --- | --- | --- |
| I would like to use **[***the new pH Strips***]** if I need to perform an anaemia test | 1 | 2 | 3 | 4 | 5 | 6 | 7 |

* See for reference– Lee, S. and Koubek R. J. (2010). Understanding user preferences based on usability and aesthetics before and after actual use.

1. **Your likelihood to recommend [***the new pH Strips***]****

| ***Instructions*** mark one box from 10 (**Extremely like)** to 0 (**Not at all likely)**  How likely is it that you would recommend to your hospital and your colleagues the use of the **[***the new pH Strips***]** to check the placement of NG tube? | | | | | | | | | | | | |
| --- | --- | --- | --- | --- | --- | --- | --- | --- | --- | --- | --- | --- |
| Extremely  likely | **10** | **9** | **8** | **7** | **6** | **5** | **4** | **3** | **2** | **1** | **0** | Not at all likely |
|  |  | |  | |  | | | | | | |  |
|  | 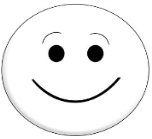 | | 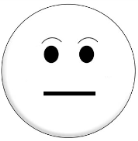 | | 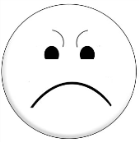 | | | | | | |  |

** See for reference - Reichheld, F. (2003). One Number You Need to Grow.
